# Synthesis of Novel Dental Nanocomposite Resins by Incorporating Polymerizable, Quaternary Ammonium Silane-Modified Silica Nanoparticles

**DOI:** 10.3390/polym13111682

**Published:** 2021-05-21

**Authors:** Alexandros K. Nikolaidis, Elisabeth A. Koulaouzidou, Christos Gogos, Dimitris S. Achilias

**Affiliations:** 1 Division of Dental Tissues’ Pathology and Therapeutics (Basic Dental Sciences, Endodontology and Operative Dentistry), School of Dentistry, Aristotle University Thessaloniki, 541 24 Thessaloniki, Greece; koulaouz@dent.auth.gr (E.A.K.); gogos@dent.auth.gr (C.G.); 2 Laboratory of Polymer and Color Chemistry and Technology, Department of Chemistry, Aristotle University Thessaloniki, 541 24 Thessaloniki, Greece; axilias@chem.auth.gr

**Keywords:** dental nanocomposite resins, dental materials, nanosilica, organosilanes, quaternary ammonium compounds

## Abstract

Diverse approaches dealing with the reinforcement of dental composite resins with quaternary ammonium compounds (QAC) have been previously reported. This work aims to investigate the physicochemical and mechanical performance of dental resins containing silica nanofillers with novel QAC. Different types of quaternary ammonium silane compounds (QASiC) were initially synthesized and characterized with proton nuclear magnetic resonance (^1^H-NMR) and Fourier transform infrared (FTIR) spectroscopy. Silica nanoparticles were surface modified with the above QASiC and the structure of silanized products (S.QASiC) was confirmed by means of FTIR and thermogravimetric analysis. The obtained S.QASiC were then incorporated into methacrylate based dental resins. Scanning electron microscopy images revealed a satisfactory dispersion of silica nanoclusters for most of the synthesized nanocomposites. Curing kinetics disclosed a rise in both the autoacceleration effect and degree of conversion mainly induced by shorter QASiC molecules. Polymerization shrinkage was found to be influenced by the particular type of S.QASiC. The flexural modulus and strength of composites were increased by 74% and 19%, while their compressive strength enhancement reached up to 19% by adding 22 wt% S.QASiC nanoparticles. These findings might contribute to the proper design of multifunctional dental materials able to meet the contemporary challenges in clinical practice.

## 1. Introduction

In the mid-1960s, dental composite resins were introduced as anterior teeth restorative materials by replacing amalgam due to aesthetic reasons, absence of mercury toxicity, low corrosion phenomena, and high adhesive ability [1,2]. From the chemical point of view, they usually consist of an organic phase comprising the basic monomers 2,2-bis[p-(2′-hydroxy-3′-methacryloxypropoxy)phenylene]propane (Bis-GMA) or 1,6-bis(methacryloxy-2-ethoxycarbonylamino)-2,4,4-trimethylhexane (UDMA) and the co-monomer triethylene glycol dimethacrylate (TEGDMA), as well as inorganic reinforcing fillers (e.g., glass, quartz, silica particles) and organosilane coupling agents binding the polymeric matrix with inorganic particles. Nevertheless, extreme oral environment conditions involving mainly complicated masticatory forces [3], pH variations [4], and dental plaque formation [5,6,7] always compose an unstable regime for dental composite resins’ elongated performance. Consequently, dental composite resins are constantly challenged with high mechanical stresses, polymerization shrinkage [8], water sorption [9], discoloration [10], and more specifically with microbial attacks [11] which can compromise their clinical longevity.

Benefits arising from the nanotechnology field may serve a variety of modern dental applications [12]. In this framework, silica nanoparticles (SNPs) are often used as reinforcing agents in dental nanocomposite resins due to their advantageous features, such as non-cytotoxicity, low cost, high stability, and their capability of being organomodified with silanes having diverse functional groups [13,14]. Much attention has been paid by several researchers dealing with the comprehension of the effect of the silica filler’s size, mass fraction, organosilane chemistry, and coupling agent content of nanosilica on the ultimate mechanical [15,16,17,18], physicochemical [19,20,21,22,23], optical [24,25], and thermal properties [21,22,23,26] of dental nanocomposite resins. In particular, Habib et al. achieved the maximum flexural properties when they used 70 wt% low dispersity silica particles of 350, 500, and 1000 nm modified with 3-(trimethoxysilyl)propyl methacrylate (γ-MPS), while the reduction of the filler size improved the optical properties and cure of depth for the produced composites [15]. Rezvani et al. found that when only 5 wt% of 10 nm silica nanoparticles were incorporated into experimental dental fiber-reinforced resins, the flexural modulus and strength could reach up to 22 GPa and 125 MPa respectively [17]. Moreover, Karabela showed that the addition of nanosilica functionalized with γ-MPS leads to lower sorption and solubility in ethanol/water solution than other silanes, such as a urethane dimethacrylate silane or the octyltrimethoxysilane (OTMS) [20]. Rodrígues proved that the spheroidal or irregular shape of silanized silica nanoclusters might influence not only the rheological, but also the mechanical and sorption properties of the dental composites [22]. However, no further information can be drawn from these literature sources in terms of any antimicrobial capability of the produced dental resins, even if it is crucial in clinical practice.

On the other hand, diverse dental materials containing quaternary ammonium compounds (QAC) have been extensively studied in regard to their outstanding antibiotic attitude against those bacteria forming dental plaque [27,28,29,30,31,32,33,34,35,36,37]. Particularly, quaternary ammonium dimethacrylates like bis(2-methacryloyloxyethyl)dimethyl ammonium bromide [38,39,40], and monomethacrylates such as dimethylaminododecyl methacrylate [41,42], dimethylaminohexadecyl methacrylate [42,43], dimethylaminopropyl methacrylate, dimethylaminohexyl methacrylate, and dimethylaminooctadecyl methacrylate [42], have been synthesized and then incorporated into bioactive Bis-GMA/TEGDMA nanocomposites in the presence of individual amorphous calcium phosphate nanoparticles (NACP) [39,41,42,43] or NACP and silver nanoparticles [38,40], leading to antimicrobial activity against *Streptococcus mutans* (*S. mutans*) [38,39] and dental plaque microcosm biofilm [40,41,42,43], without jeopardizing the mechanical properties in most cases. Furthermore, restorative composite resins filled with quaternary ammonium polyethyleneimine (QPEI) nanoparticles also showed a significant antibacterial effect against *S. mutans* [44,45]. Furthermore, the separate insertion of silica particles and monomer 12-methacryloyloxydodecylpyridinium (MDBP) into dental resins, resulted in the inhibition of *S.mutans* [46], while the release attitude of unpolymerized MDBP on bacterial growth was also investigated [47]. Despite this, all studies dealing with the utilization of individual QAC monomers provided insufficient experimental data regarding the overall physicochemical behavior of the ultimate dental composite resins.

A new and promising approach towards a more stable immobilization of the biocide into the dental composite resins could encompass the incorporation of silica nanoparticles directly functionalized with QAC. To the best of our knowledge, limited reports negotiate the reinforcement of dental composite resins with such potential antibacterial agents, while the mechanical and physicochemical attitudes of those dental nanocomposites have not been comprehensively studied so far. Indeed, Makvandi et al. described the remarkable antimicrobial activity against *S.mutans* for Bis-GMA/TEGDMA nanocomposite resins filled with 2.5–10 wt% bifunctional silanized silica nanoparticles containing glycidyltrimethyl ammonium segments and methacrylic functionality [48]. However, further investigation of the above materials was restricted to the evaluation of flexural properties without looking for any findings regarding polymerization kinetics and degree of conversion, shrinkage, sorption, or solubility. On the other hand, antimicrobial effects against *Enterococcus faecalis* bacteria were also induced when Zaltsman functionalized silica particles with 170 dimethyloctyl ammonium groups per nm^2^ were subsequently inserted in typical Bis-GMA/UDMA/TEGDMA dental restorative composites (2 wt% filler loading, 186 nm average diameter) [49]. Nevertheless, no other available data arose dealing with the physical, chemical or mechanical properties of the aforementioned dental composites.

The objective of the present work is the synthesis of new experimental dental nanocomposite resins by incorporating novel bifunctional silane-modified silica nanoparticles. Based on the concept associated with the previously described QAC’s antimicrobial activity, new polymerizable quaternary ammonium silane compounds (QASiC) were initially synthesized, then used to modify silica nanoparticles, and the obtained organomodified nanosilica (S.QASiC) was finally inserted into dental nanocomposite resin formulations. The effect of the QASiC structure on the silica silanization process, as well as the influence of the different type of methacrylated S.QASiC in the physicochemical and mechanical performance of the produced nanocomposites were extensively studied. The significance of the current research lies in the prospect of its further contribution to the designation of modern dental materials, as it generates prerequisite data on physicochemical and mechanical attitudes which can be further combined with prospective antibacterial and cytotoxicity studies towards a holistic approach capable of meeting the requirements in clinical practice.

## 2. Materials and Methods

### 2.1. Materials

The totality of chemical substances used for the purposes of this study is listed in Table 1.

### 2.2. Synthesis of Nanosilica Organomodifiers (QASiC)

**Scheme 1:**
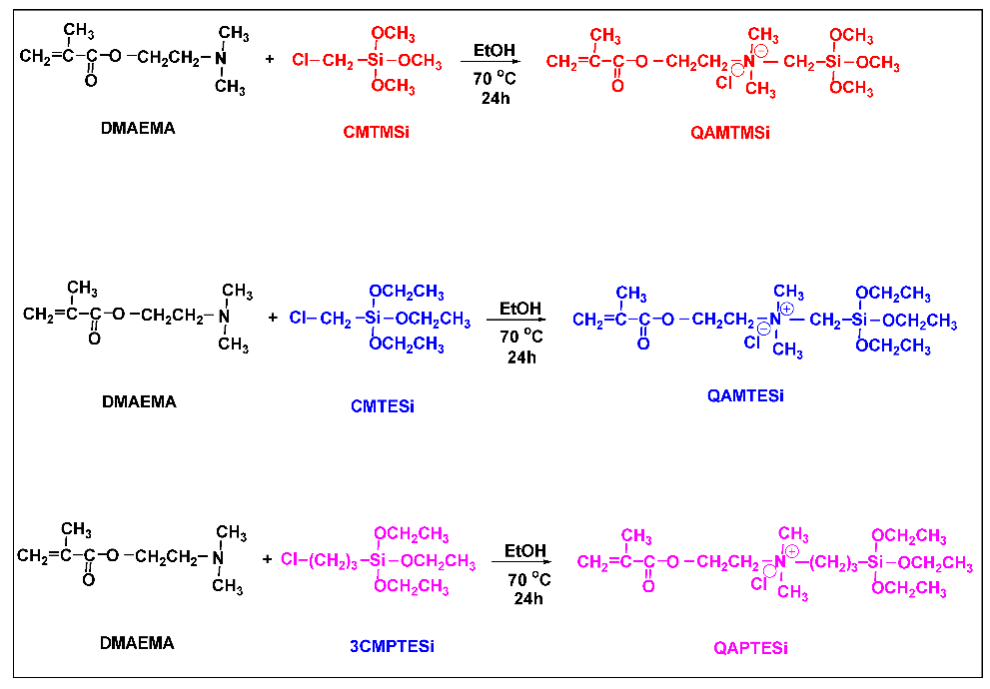
Modified Menschutkin reactions applied for QASiC synthesis.

The procedure followed for synthesis of the new methacrylated quaternary ammonium silanes is a variant of Menschutkin reaction [50]. Briefly, 10 mmol of DMAEMA and 10 mmol of CMTMSi, or CMTESi, or 3CPTESi were mixed with 3 g of ethanol in a 20 mL scintillation vial and the reaction was carried out at 70 °C for 24 h. The solvent was then evaporated under vacuum at 50 °C to obtain the white crystals of QAMTMSi, QAMTESi, and QAPTESi products respectively (Scheme 1).

### 2.3. Surface Modification of Nanosilica with Quaternary Ammonium Silane Coupling Agents 

The minimum weight percent of silane theoretically needed for the silica silanization was calculated according to the following equation [51,52]:

(1) $$\mathrm{Organosilane}\;\left(\mathrm{wt}\%\right)=\frac{\mathrm{Filler}\;\mathrm{surface}\;\mathrm{area}\;\left(m^{2}\cdot g^{-1}\right)}{\mathrm{Silane}\;\mathrm{surface}\;\mathrm{coverage}\;\left(m^{2}\cdot g^{-1}\right)}\times 100$$

Taking into account a nanosilica with 200 m^2^·g^−1^ specific surface area and γ-MPS molecules with 2525 m^2^·g^−1^ surface coverage [53], an 8 wt% amount of γ-MPS relative to silica was required for a complete silanization. Given that the molecular weights of the prepared silanes QAMTMSi, QAMTESi, and QAPTESi (327.5, 369.5, and 397.5) were much higher than that of γ-MPS (248.3), the weight percent required for the minimum coverage of the silica should be lower than 8 wt%. However, the silane amount of 10 wt% relative to silica was considered to ensure a complete coverage of silica surface by silane [20].

**Scheme 2:**
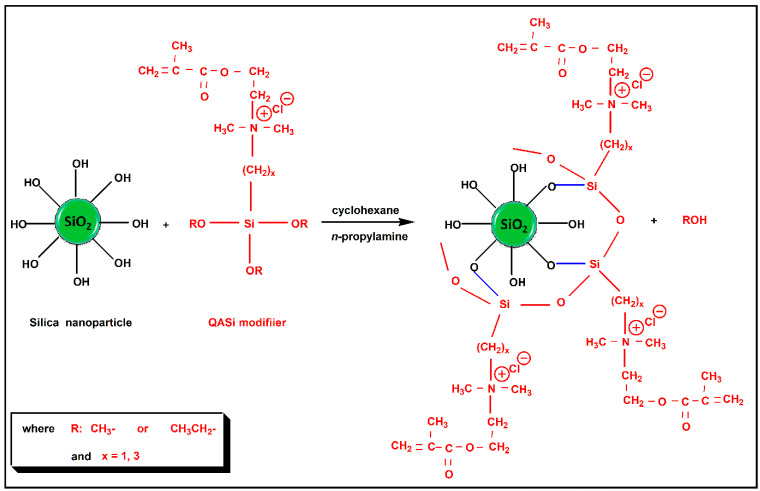
Silanization process involving silica nanoparticles and different QASiC modifiers.

The silica nanoparticles were silanized on the basis of the Chen and Brauer technique [54], by using the previously prepared silanes QAMTMSi, QAMTESi and QAPTESi (Scheme 2). Briefly, the nanosilica (5.0 ± 0.05 g), the organosilane (0.50 ± 0.01 g), cyclohexane (100 mL) and n-propylamine (0.1 ± 0.01 g) were mixed using a mechanical stirrer at room temperature for 30 min and then at 60 °C for 30 min. The solvent and volatile by-products were then removed at 60 °C by means of a rotary evaporator. The organically modified silica nanopowder (S.QAMTMSi, S.QAMTESi, S.QAPTESi) was further heated at 100 °C for 1h in the rotary evaporator and finally dried at 80 °C in a vacuum oven for 20 h. The same experimental procedure was followed by using the widely utilized γ-MPS silanization agent to obtain a control sample of surface silanized nanosilica (S.MPS).

### 2.4. Preparation of the Uncured Dental Composite Pastes

Five groups of experimental composites were prepared by initially mixing a Bis-GMA/TEGDMA base (50:50 wt/wt) which contained camphorquinone (0.2 wt%) and DMAEMA (0.8 wt%) as a photo-initiating system. Afterward, the neat nanosilica, S.MPS, S.QAMTMSi, S.QAMTESi, and S.QAPTESi nanoparticles were individually inserted in the resin by manual mixing until the powder was completely wetted with organic matrix, and the obtained mixture was ultrasonicated for 10 min. The nanofiller loading was determined to 22 wt% to ensure paste handling properties almost similar to a commercial dental composite resin. Bis-GMA/TEGDMA pure matrix was also prepared to be used as control material.

### 2.5. Measuremens

#### 2.5.1. Structural Characterization of QASiC

Proton nuclear magnetic resonance (^1^H-NMR) measurements were carried out in dimethyl sulfoxide-d_6_ using a BrukerAV-250 spectrometer (Bruker Analytik GmbH, Rheinstetten, Germany) at the resonance frequency of ^1^H-250 MHz. Data were collected and processed using a TopSpin 2.1 software provided by Bruker. 

Both QASiC and their corresponding starting materials were scanned by means of a Spectrum One Perkin-Elmer FTIR spectrometer (PerkinElmer Inc., Waltham, MA, USA). A spot of samples was placed between two NaCl crystals and spectra were obtained over the 4000–500 cm^−1^ region and acquired with a resolution of 4 cm^−1^ and a total of 32 scans per spectrum. A commercial software Spectrum v5.0.1 (Perkin-Elmer LLC 1500F2429) was used to acquire and process and calculate all the data from the spectra.

#### 2.5.2. Characterization of the Organically Modified Nanosilica

KBr disks of the obtained silica nanopowders were initially prepared by means of a manual hydraulic press and then measured by using a Spectrum One Perkin-Elmer FTIR spectrometer (PerkinElmer Inc., Waltham, MA, USA), in the scanning range of 4000–600 cm^−1^. The resolution of the equipment was 4 cm^−1^. A commercial software Spectrum v5.0.1 (Perkin-Elmer LLC 1500F2429) was used to process and calculate all the data from the spectra.

Thermogravimetric analysis (TGA) was performed with a SETARAM SETSYS TG-DTA 16/18 instrument (Setaram instrumentation, Lyon, France). The Calisto program was employed to collect and process the data. The samples (8 ± 0.2 mg) were placed in 170 μL alumina crucibles, while a blank measurement was performed and subsequently was subtracted by the experimental curve, in order to eliminate the buoyancy effect. Nanosilica powder samples were heated from ambient temperature up to 750 °C in a 10 mL·min^−1^ N_2_ flow at the heating rate of 10 °C min^−1^. Continuous recording of both sample temperature and sample weight was carried out. Thermal degradation onsets were taken from the initial peak of the derivative of the TGA curve across the mass loss transition.

#### 2.5.3. Surface Morphology Measurements of Nanocomposite Resins

Scanning electron microscopy (SEM) was carried out using a JEOL JSM-6390LV (JEOL USA, Inc., Peabody, MA, USA) scanning microscope (0.5–30 kV) with a high resolution of 3 nm equipped with an energy-dispersive X-ray (EDX) INCAPentaFETx3 (Oxford Instruments, Abingdon, UK) microanalytical system. All the studied surfaces were coated with carbon black to avoid charging under the electron beam. The samples were probed with a beam of electrons focused into a spot on the sample surface and Smile Shot^TM^ software was used to capture the microphotos.

#### 2.5.4. Physicochemical Properties 

Polymerization shrinkage kinetics was conducted according to the “bonded-disk” method which was initially published and further refined by Watts and co-workers [55,56,57]. Briefly, a disk-shaped unset specimen with dimensions of 1.0 mm × 8.0 mm (height × diameter) was formed and centrally positioned upon a 3-mm thick rigid glass plate. A flexible cover-slip diaphragm, supported by an outer peripheral brass ring with internal diameter circa 15 mm, was rested on the upper surface of the specimen disk so as to be adherent. A uniaxial LVDT (linear variable displacement transducer) measuring system was positioned centrally onto the cover slip. The signal from the LVDT was transmitted to a computer by a transducer indicator (E 309, RDP Electronics Ltd., Wolverhampton, UK), and a high-resolution analog to digital converter (ADAM-4016 acquisition module).The data acquisition was supported by the datalogger software AdvantechAdam/Apax.NET Utility, version 2.05.11. Measurements records were taken by continuous irradiation of specimens with a LED polymerization unit (Bluephase^®^ Style M8, IvoclarVivadent AG, FL-9494, Schaan, Liechtenstein) at 800 mW·cm^−2^ ± 10% for 5 min directly from beneath the glass plate at room temperature. A radiometer (Hilux, Benlioglu Dental Inc., Ankara, Turkey) was used to verify the output irradiance of the light-curing device. Five repetitions (*n* = 5) were made at each specimen. Strain was calculated as:

(2) $$\varepsilon \left(\%\right)=100\times \frac{\Delta L}{L_{0}}$$

where ε (%) represents the strain (%), DL and L_0_ are the shrinkage displacement and the initial specimen thickness, respectively.

Polymerization kinetics was performed by placing a small amount of each composite between two translucent Mylar strips, which were pressed to produce a very thin film. The films of unpolymerized composites were exposed to visible light as previously described, and immediately scanned by a Spectrum One Perkin–Elmer FTIR spectrometer (PerkinElmer Inc., Waltham, MA, USA) at different curing time intervals (0, 5, 10, 15, 20, 25, 30, 40, 60, 80, 120, 180 s). A spot of samples was placed between two NaCl crystals and spectra were obtained over the 4000–600 cm^−1^ region and acquired with a resolution of 4 cm^−1^ and a total of 32 scans per spectrum. Commercial software Spectrum v5.0.1 (Perkin-Elmer LLC 1500F2429) was used to process and calculate all the data from the spectra.

The area of aliphatic C=C peak absorption at 1637 cm^−1^, and the aromatic C=C peak absorption at 1580 cm^−1^ were determined, utilizing a base line technique which proved the best fit to the Beer–Lambert law [58]. The aromatic C=C vibration was used as an internal standard. The percent degree of monomer conversion (DC%) of the cured specimen, which expresses the percent amount of double carbon bond that reacted at each time period, was determined according to the equation:

(3) $$\mathrm{DC}\left(\%\right)=\left[1-\frac{{\left(\frac{A_{1637}}{A_{1580}}\right)}_{\mathrm{polymer}}}{{\left(\frac{A_{1637}}{A_{1580}}\right)}_{\mathrm{monomer}}}\right]\times 100$$

#### 2.5.5. Mechanical Properties 

For flexural tests, bar-specimens were prepared by filling a Teflon mold (2 mm × 2 mm × 25 mm) with unpolymerized paste in accordance with ISO 4049. The mold surfaces were overlaid with glass slides covered with a Mylar sheet to avoid air entrapping and adhesion of the final set material. The assembly was held together with spring clips and irradiated by overlapping on both sides, as previously described. Each overlap irradiation lasted for 40 s. Ten specimen bars (*n* = 10) were prepared for each nanocomposite. The specimens were stored in water for 24 h at 37 °C after curing. Afterward, they were bent in a three-point transverse testing rig with 20 mm between the two supports (3-point bending). The rig was fitted to a universal testing machine (Testometric AX, M350-10kN, Testometric Co. Ltd., Rochdale, UK). All bend tests were carried out at a cross-head speed of 0.5 mm·min^−1^ until fracture occurred. The load and the corresponding deflection were recorded. The data were collected and processed by means of the software WinTestAnanlysis CX Version 3.5.30.10. The flexural modulus (E) in GPa and flexural strength (σ) in MPa were calculated according to the following equations:

(4) $$E=\frac{F_{1}l^{3}}{4{\mathrm{bd}}_{1}h^{3}}10^{-3}\;\mathrm{and}\;\sigma =\frac{3{\;F}_{\max}\;l}{2{\mathrm{bh}}^{2}}$$

where: F_1_ is the load recorded in N, F_max_ is the maximum load recorded before fracture in N, l is the span between the supports (20 mm), b is the width of the specimen in mm, h is the height of the specimen in mm, and d_1_ is the deflection (in mm) corresponding to the load F_1_.

Cylindrical specimens (4 × 6 mm, diameter × height) for compressive tests were prepared (*n* = 10), under curing and storing as described above. Test specimens were fractured on a universal testing machine (Testometric AX, M350-10kN, Testometric Co. Ltd., Rochdale, UK). All measurements were carried out at a cross-head speed of 1 mm·min^−1^ until fracture occurred. The data were acquired and processed by means of the software WinTestAnanlysis CX Version 3.5.30.10. The maximum load was recorded and the compressive strength (σ) in MPa was calculated using the standard formula:

(5) $$\sigma =\frac{4F}{{\pi D}^{2}}$$

where F is the maximum load (N) and D is the specimen diameter (mm).

#### 2.5.6. Statistical Analysis

One-way ANOVA test followed by Tukey–Kramer multiple comparisons test was applied for the obtained flexural modulus, flexural strength and compressive strength parameters. The values of the measured mechanical properties represent mean values ± standard deviation of replicates (*n* = 10) for flexural modulus, while median values ± interquartile range (IQR) for flexural and compression strength.

## 3. Results and Discussion

### 3.1. ^1^H-NMR and FTIR Data of the Synthesized QASiC

**Figure 1:**
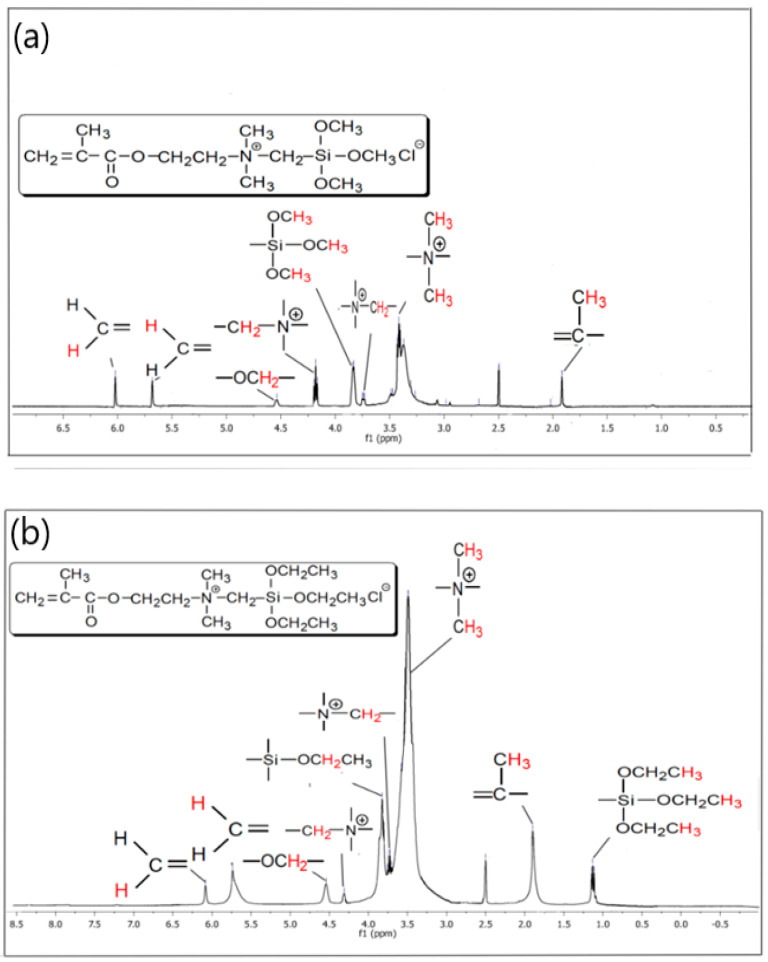

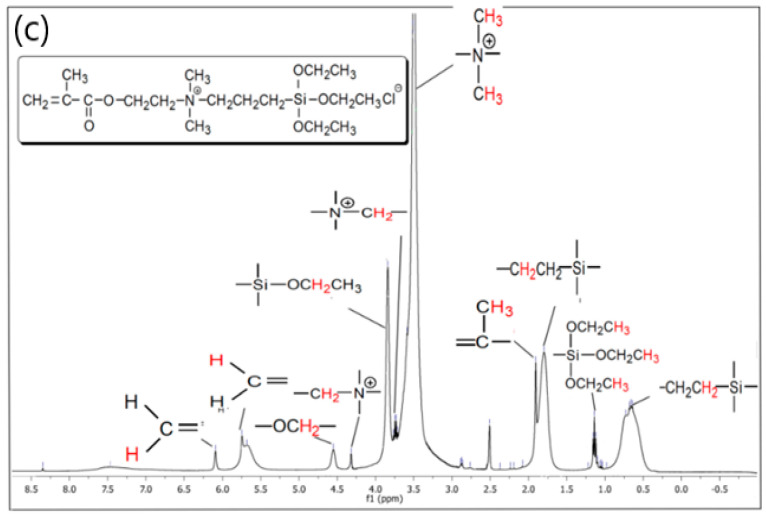
^1^H-NMR spectra of the: (**a**) QAMTMSi; (**b**) QAMTESi; (**c**) QAPTESi organosilanes.

The structures of the above compounds were identified by the following ^1^H-NMR [δ (ppm)] peak assignments: a) QAMTMSi: 6.02 (1H, m, CH_2_=), 5.68 (1H, m, CH_2_=), 4.54 (2H, w, –OCH_2_–), 4.18 (2H, m, –CH_2_–N+), 3.83 (9H, m, -Si(OCH_3_)_3_), 3.74 (2H, w, N^+^–CH_2_–), 3.47 (6H, s, N^+^–(CH_3_)_2_), 1.91 (3H, m, CH_3_–C=), b) QAMTESi: 6.07 (1H, w, CH_2_=), 5.68 (1H, m, CH_2_=), 4.55 (2H, w, –OCH_2_–), 4.30 (2H, v.w, –CH_2_–N^+^), 3.84 (6H, s, Si(O–CH_2_–CH_3_)_3_, 3.70 (2H, w, N^+^–CH_2_–), 3.49 (6H, v.s, N^+^–(CH_3_)_2_), 1.91 (3H, s, CH_3_–C=), 1.10 (9H, m, Si(OCH_2_–CH_3_)_3_), and c) QAPTESi: 6.09 (1H, w, CH_2_=), 5.68 (1H, m, CH_2_=), 4.55 (2H, w, –OCH_2_–), 4.32 (2H, v.w, –CH_2_–N^+^), 3.84 (6H, s, Si(O–CH_2_–CH_3_)_3_, 3.71 (2H, w, N^+^–CH_2_–), 3.50 (6H, vs., N^+^–(CH_3_)_2_), 1.91 (3H, s, CH_3_–C=), 1.80 (2H, s, –CH_2_–CH_2_Si), 1.10 (9H, m, Si(OCH_2_–CH_3_)_3_), 0.66 & 0.64 (2H, m, –CH_2_–Si). Representative ^1^H-NMR spectra for QAMTMSi and QAPTESi products are illustrated in Figure 1.

**Figure 2:**
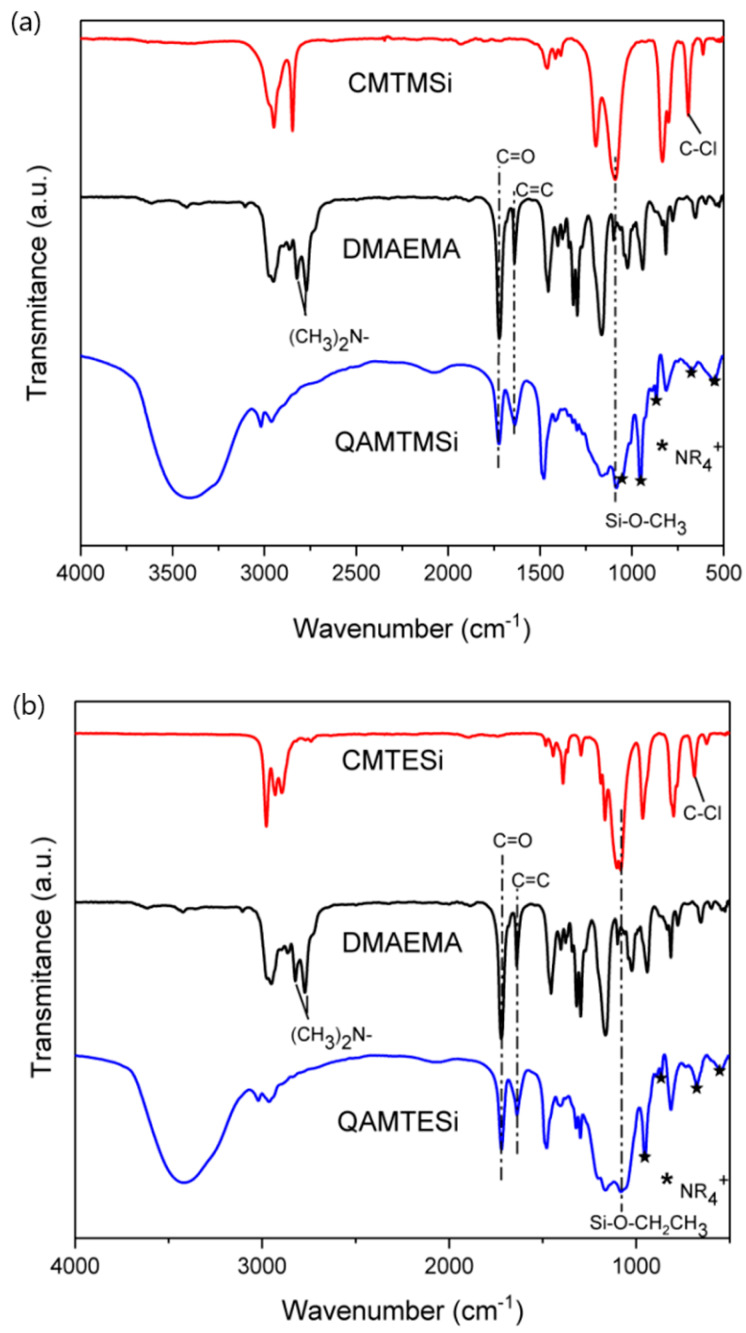

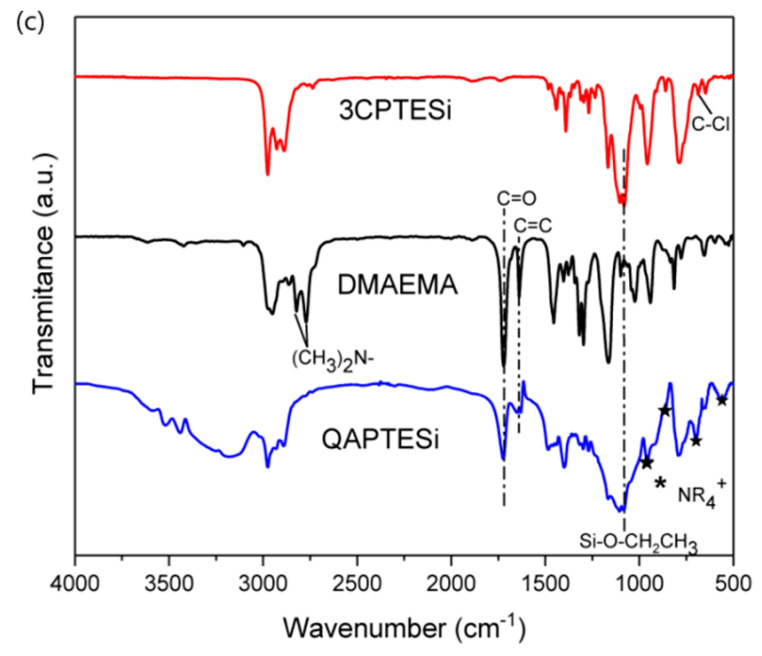
FTIR spectra of the synthesized: (**a**) QAMTMSi; (**b**) QAMTESi; (**c**) QAPTESi organosilanes accompanied by the corresponding of their starting compounds. Asterisk symbols stand for the NR4+ groups of the obtained QASiC.

**Figure 3:**
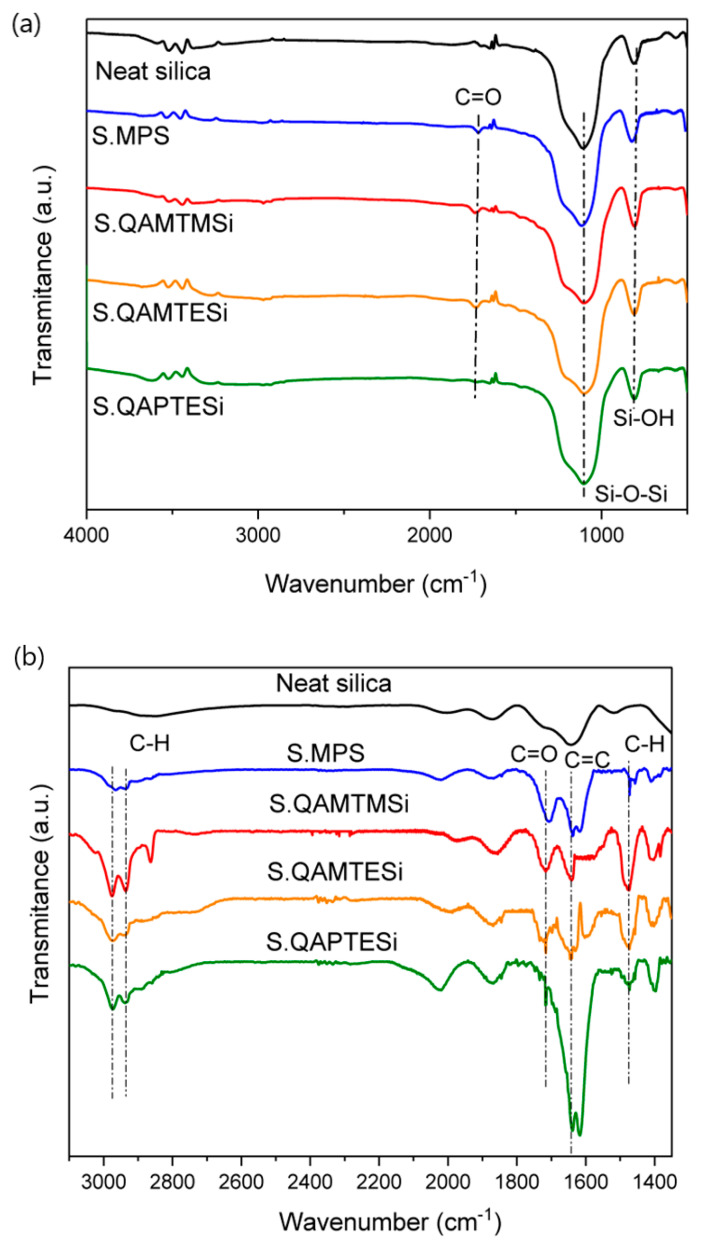
Comparative FTIR spectra in the scanning ranges of: (**a**) 4000–500 cm^−1^; (**b**) 3200–1350 cm^−1^ between neat and diverse organomodified silica nanoparticles.

The FTIR spectra recorded for the newly synthesized QASiC compounds along with their corresponding starting materials are represented in Figure 2. The absorption peaks at 3020 and 2959 cm^−1^ due to =CH and C–H stretch vibrations occurred in all spectra of the obtained products QAMTMSi (Figure 2a), QAMTESi (Figure 2b) and QAPTESi (Figure 2c), denoting the existence of CH, CH_2_ and CH_3_ groups [59]. The characteristic bands at 2822 and 2771 cm^−1^ found in DMAEMA spectrum corresponded to the C–H stretching of (CH_3_)_2_N– groups. However, these specific bands were not detected in the spectra of the produced organosilanes. The two absorption peaks at 1722 and 1640 cm^−1^ observed in all QASiC modifiers were assigned to C=O and C=C stretch vibrations respectively, as a result of the DMAEMA segments contained in their structures. Moreover, the common absorption peaks at 1088 cm^−1^ recorded in each QASiC spectrum were attributed to Si–OCH_3_ and Si–OCH_2_CH_3_ groups coming from CMTMSi (Figure 2a), CMTESi (Figure 2b) and 3CPTESi (Figure 2c) respectively. In each case, Figure 2 disclosed four new bands, namely at 952, 864, 677, and 511 cm^−1^, which could be assigned to the possible formation of new NR_4_^+^ complexes, given that NR_4_^+^ groups are well-known to absorb in the range of 1100–450 cm^−1^ [28]. In addition, although the band observed at 690 cm^−1^ in the CMTMSi, CMTESi, and 3CPTESi spectra could be assigned to C–Cl bending, it was not detected for the QASiC modifiers any longer.

Herein, three new methacrylated quaternary ammonium silanes were synthesized on the basis of the Menschutkin reaction between a tertiary amine-group and an organo-halide, also known concerning the high yields of pure products [28,38,41]. Scheme 1 illustrates a modified model of Menschutkin reaction where the DMAEMA played the role of the tertiary amine, whereas the organosilanes CMTMSi, CMTESi, and 3CPTESi could act as organo-halides. As described above, the combination of ^1^H-NMR and FTIR findings confirmed the successful synthesis of QAMTMSi, QAMTESi, and QAPTESi organo-silanes.

### 3.2. FTIR Analysis of Silica Nanoparticles

Figure 3a displays the FTIR measurements taken for neat silica, S.MPS, S.QAMTMSi, S.QAMTESi, and S.QAPTESi nanoparticles in the wide scanning range of 4000–500 cm^−1^. The strongest peaks at 1100 and 810 cm^−1^ appearing in all presented silica spectra can be assigned to Si-O-Si and Si-OH vibrations, respectively, originating from the non-treated nanosilica. However, it is obvious that the high intension peaks due to the abundance of siloxane and silanol groups extremely attenuated the signal of other possible absorption bands. A further scanning in the narrower range of 3200–1350 cm^−1^ (Figure 3b) enhanced the occurrence of additional peaks at 2970 and 2930 cm^−1^ due to C-H stretching, at 1720 cm^−1^ assigned to C=O groups, and finally at 1640 cm^−1^ and 1472 cm^−1^ related to C=C stretching and C-H bending vibrations correspondingly. In each case, the presence of the above absorptions is correlated with QASiC structures, thus implying the accomplishment of the silanization reaction regardless of the particular type of organosilane.

### 3.3. Thermogravimetric Analysis (TGA) of Silica Nanoparticles

**Figure 4:**
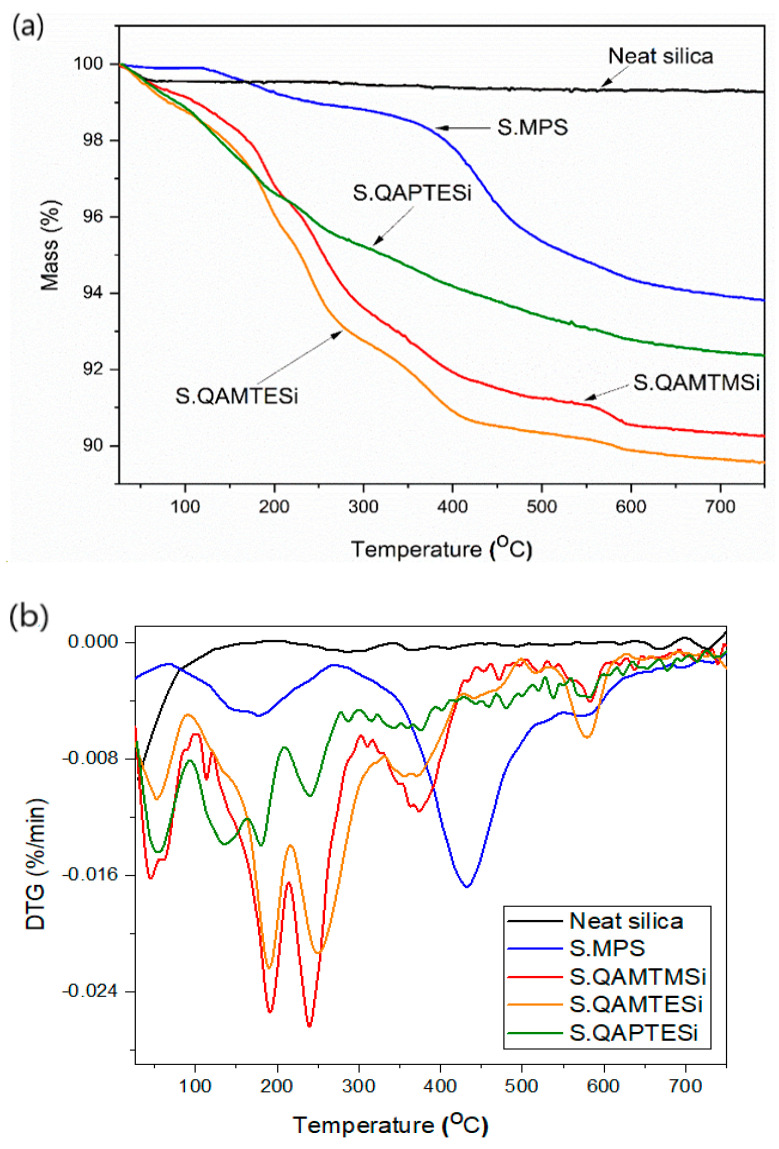
(**a**) TG comparative scans reflecting the different thermal stability of the tested silica nanoparticles; (**b**) DTG curves for neat silica, S.MPS, S.QAMTMSi, S.QAMTESi, S.QAPTESi.

The TG plots used to determine the mass loss of the different organically modified silica nanoparticles are illustrated in Figure 4a. It is apparent that their thermal stability varies according to their particular organosilane content. Higher mass loss was observed for the total of silane modified nanosilicas when compared to neat silica particles, due to the presence of thermally labile organic molecules on their surface. The different thermal attitude of each type of silica not only supports the FTIR findings regarding the successful silanization of silica, but also reflects the diversity of the silane content. Typical differential thermogravimetric analysis (DTG) curves (Figure 4b) drawn for the studied nanoparticles revealed a two-step thermal degradation for S.MPS, while a multi-step decomposition process is associated with S.QAMTMSi, S.QAMTESi, and S.QAPTESi. Mass loss in the range of 50–180 °C [51] or even more at ~200 °C [21] has been related to the removal of physically absorbed organosilanes. These phenomena seem to dominate up to the maximum degradation rate of 190 °C, followed by the removal of the chemically bonded QASiC molecules, and finally the condensation of the surface silanol groups corresponding to a weaker mass loss (Figure 4b) [51].

**Scheme 3:**
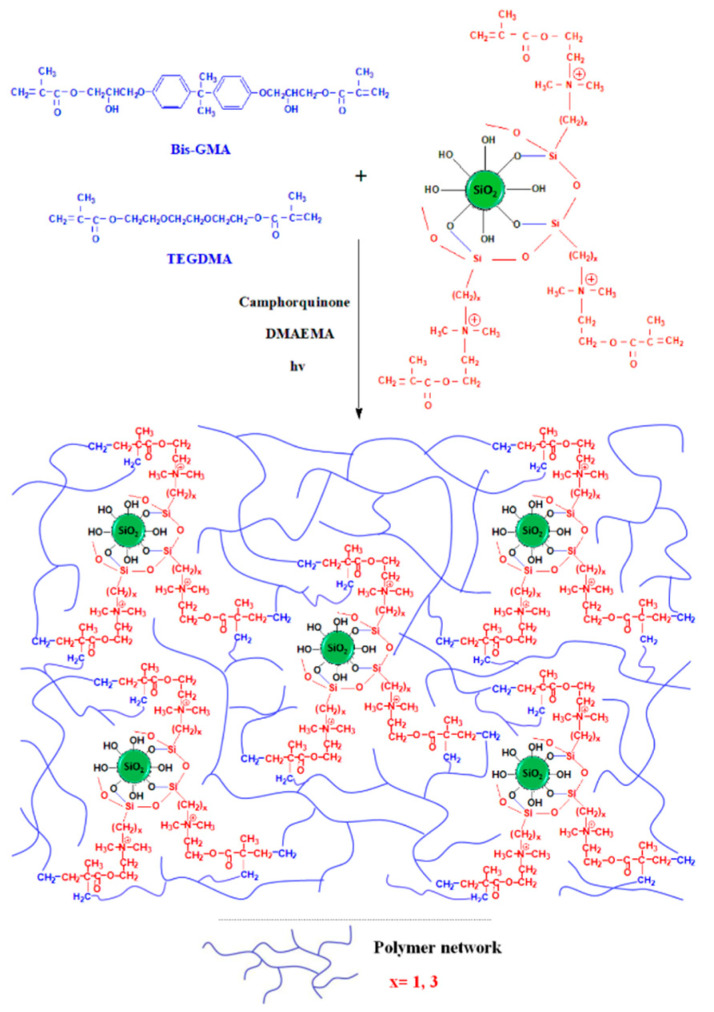
Proposed illustration of the nanocomposite network formation mechanism showing the potential chemical interactions between organomodified silica nanofillers and dental resin monomers.

The wt% values representing the total QASiC content as well as the physical or chemical aliquots attached on the silica surface are listed in Table 2. It can be seen that S.QAMTMSi exhibits the highest chemical binding (5.43 wt%) while the longer chains of S.QAPTESi characterized by lower mobility rather do not favor the surface attachment (3.44 wt%). It is worth pointing out that silica absorbed more S.QAMTESi in a physical way (4.50 wt%) than S.QAMTMSi (3.55 wt%), whereas a lower portion of S.QAMTESi was chemically attached (5.27 wt%) in comparison to S.QAMTMSi (5.43 wt%), even if the ultimate chemical structure of the organomodified nanosilica remains exactly the same (Scheme 2). The above claim could be evidence that methoxy groups from S.QAMTMSi might lead to a higher extent of QASiC binding than ethoxy groups, and thus contribute to a more effective silanization process. Besides, the removal of ethanol is considered an important factor affecting the yield of the equilibrium silanization reaction [60]. On the other hand, when the organosilane reactant is used in excess, the physically absorbed silane molecules may occur in the form of an upper second layer hydrogen bonded to that of the chemically absorbed which is covalently bonded to the silica surface. Considering a possible nanocomposite structure (Scheme 3), the second layer might further affect the polymer matrix–filler linkage, resulting in composite weakness, so a functional ratio was also calculated as an index of effective matrix–filler coupling (Table 2).

### 3.4. SEM Images of Nanocomposites’ Surface

**Figure 5:**
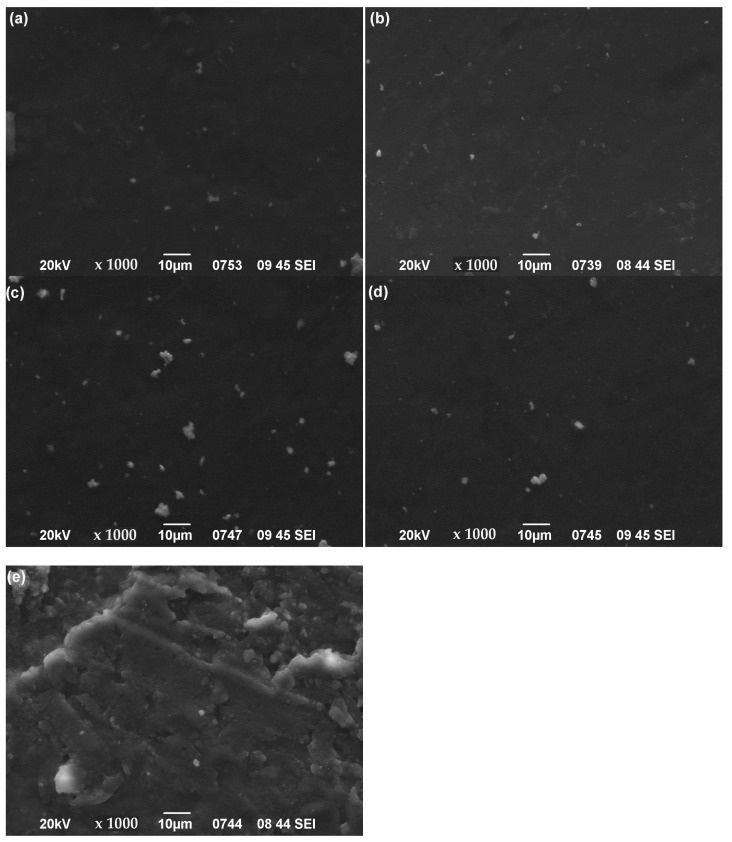
SEM microphotos taken for dental nanocomposite resins filled with 22 wt% of: (**a**) neat silica; (**b**) S.MPS; (**c**) S.QAMTMSi; (**d**) S.QAMTESi; (**e**) S.QAPTESi nanoparticles (×1000 magnification).

SEM microphotos were used to demonstrate a typical distribution of silica nanoparticles in the polymer matrix, contributing to the structural characterization of nanocomposite surface. Figure 5 shows indicative SEM micrographs of the obtained dental nanocomposite resins, where the white dots of different size represent the inorganic nanoclusters or larger silica aggregates. Nanocomposites filled with neat silica and S.MPS (Figure 5a,b) exhibited a random dispersion of small nanoclusters corresponding to relatively sufficient filler dispersion in the polymer network. Agglomerates reaching almost 8 μm size along with smaller nanoclusters occurred when S.QAMTESi nanoparticles were utilized (Figure 5d), while a greater number of microscale silica aggregates was observed when composites were loaded with 22 wt% S.QAMTMSi (Figure 5c). The co-occurrence of individual microparticles and larger aggregates was also reported by Rodriguez and co-workers [18] for dental resin composites reinforced with 20 wt% MPS-functionalized silica nanoparticles. Moreover, less homogeneous surface morphology composed of even larger agglomerates and discrete voids in polymer matrix constituted a distinguishing feature for dental nanocomposite resins containing S.QAPTESi nanofillers (Figure 5e).

### 3.5. FTIR Analysis-Degree of Conversion (DC%)

**Figure 6:**
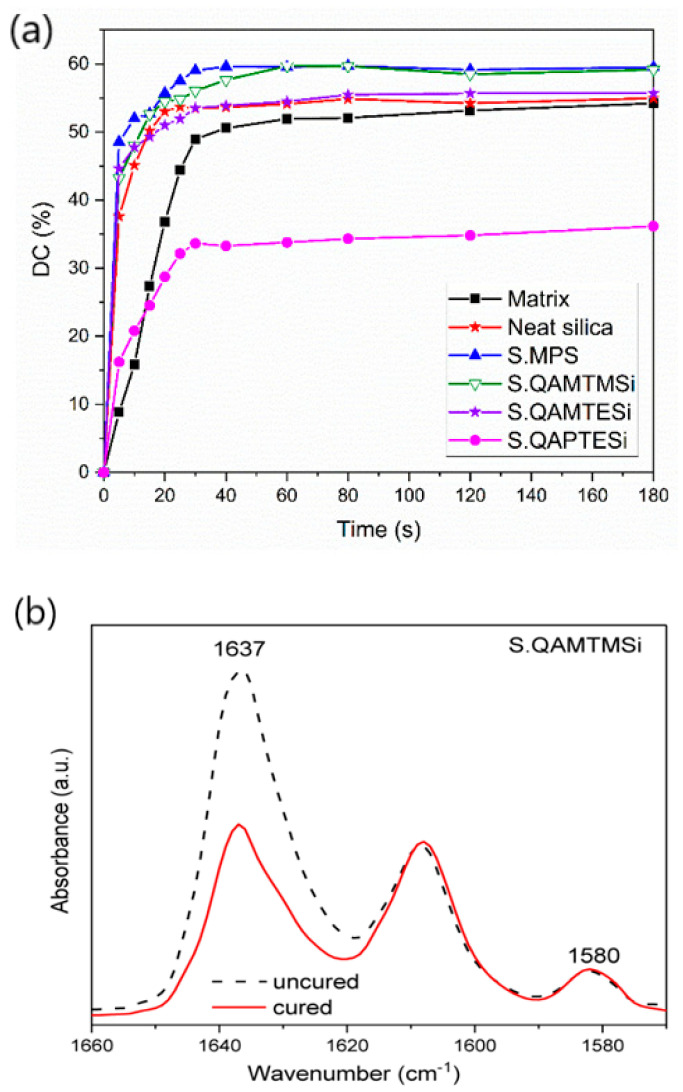

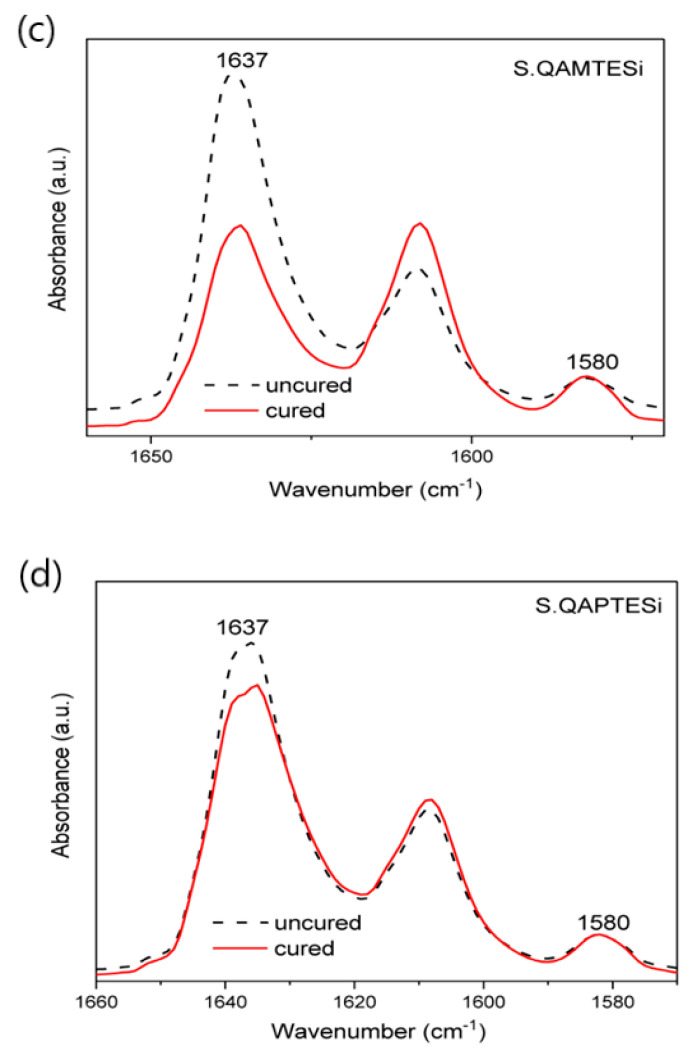
(**a**) Degree of conversion (DC%) versus curing time for the Bis-GMA/TEGDMA matrix and the experimental dental nanocomposite resins filled with 22 wt% of different type of silica nanoparicles. FTIR spectra with measured peak areas (1637 and 1580 cm^−1^) used to calculate the percent degree of conversion (DC%) for uncured and cured composites filled with: (**b**) S.QAMTMSi; (**c**) S.QAMTESi; (**d**) S.QAPTESi nanocomposites.

Polymerization kinetics plots indicating the DC (%) changes over time for Bis-GMA/TEGDMA resin and the corresponding nanocomposites, as well as the FTIR absorption peaks selected for the calculation of the experimental DC values are given in Figure 6. A steep augmentation of the DC was clearly observed five minutes after the commencement of the curing reaction for the most of the materials tested (Figure 6a). This trend was more intensive for the majority of the obtained nanocomposites in comparison to Bis-GMA/TEGDMA resin and could be attributed to the enhanced auto-acceleration or gel-effect phenomenon, due to the influence of the diffusion-controlled phenomena on the termination reaction [61]. In this state, the additional presence of silica nanoparticles may further reduce the free volume constraining the movement of live macroradicals to find each other and react, thus leading to their local concentration increase and finally to extremely high reaction rates. The above phenomenon can be amplified by the those nanofillers especially modified with the highly reactive S.MPS, S.QAMTMSi, and QAMTESi, because they could be considered relatively smaller molecules than S.QAPTESi, whereas they were found to be more chemically absorbed on silica’s surface in comparison to the QAPTESi modifier (Table 2). Hence, the S.MPS, S.QAMTMSi, and S.QAMTESi nanosilica might contribute to the higher crosslinking density of the formed network, heavily restricting the mobility of macroradicals. The significantly induced gel-effect continued until 48.5%, 43.2%, and 44.6% conversions were achieved. From this point of view, the bulkiness and lowest ratio of the chemically bonded QAPTESi deeply affected the auto-acceleration-effect up to 16.2%.

The final degree of conversion values determined for the neat Bis-GMA/TEGDMA resin and the total of the obtained dental nanocomposite resins and some representative commercially available dental composite resins are listed in Table 3. The calculated conversions for the most resins varied between 54.2% and 59.5% and were almost comparable to those found in literature for Bis-GMA/TEGDMA systems ranging from 46.84% to 75% [21,62,63,64] and higher than two typically commercial Filtek^TM^Z350 XT and Tetric^®^ N-Ceram Bulk Fill [65]. Moreover, it is clear that dental nanocomposites loaded with neat nanosilica and S.QAMTESi modified nanoparticles slightly increased the ultimate double bond conversion of the organic monomers up to 54.98% and 55.67%, respectively. On the other hand, the presence of nanofillers silanized with S.MPS and S.QAMTMSi strongly improved the DC of nanocomposites, resulting in the maximum values of 59.51% and 59.16%, correspondingly. However, when nanocomposites were filled with S.QAPTESi nanosilica, the final DC was decreased to only 36.17%. It could be assumed that the previously described rising tendencies of conversion values ascribed to auto-acceleration process were gradually moderated by the well-known glass-effect which eventually determines the final DC. Since at high conversions the propagation reaction is influenced by diffusion-controlled phenomena, the presence of nanosilica along with the developed polymer network (Scheme 3) might further act as an additional hindrance against the movement of small monomer molecules to find and react with macroradicals [61,66,67,68]. As a result, the reaction rate is zeroed easier although non-reacted monomers still remain.

### 3.6. Polymerization Shrinkage Kinetics

**Figure 7:**
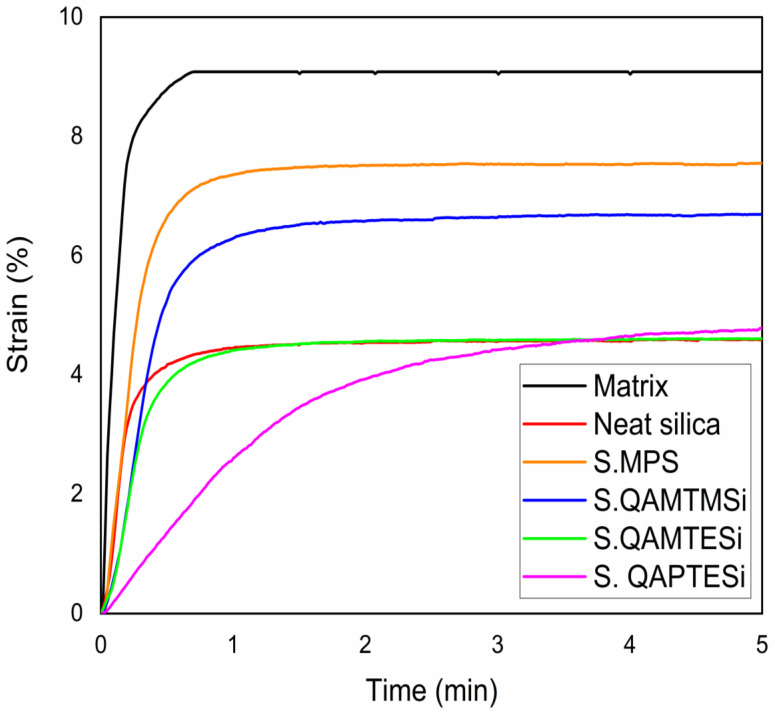
Time dependence of polymerization shrinkage strain of Bis-GMA/TEGDMA matrix and dental nanocomposite resins containing 22 wt% of diverse types of organically modified nanosilica.

Polymerization shrinkage, also known as setting contraction, relates to the volumetric reduction of composite resins due to the transformation of longer van der Waals interactions between methacrylate monomers into shorter covalent bonds in polymer matrix through the free radical polymerization process. The above alterations should be practically limited, as they are responsible for shrinkage stresses formation leading to marginal microleakage at the tooth-restoration interface [69] and finally to secondary caries and postoperative pain [70]. The volumetric contraction can be usually controlled by manipulating several parameters such as the structure and content of reactive monomers, the type and composition of reinforcing fillers, as well as the degree of double bonds conversion [71,72]. Figure 7 shows the polymerization shrinkage kinetics by plotting curves corresponding to the strain volumetric changes (%) versus setting time for the neat Bis-GMA/TEGDMA resin and the relative silica nanocomposites. The total strain values after five minutes of curing are given in Table 3. Regarding the present experimental conditions, an abrupt increase of strain was observed during the first 0.20–0.45 minfor most of the tested resins (Figure 7). Lower rates of setting contraction were revealed for dental nanocomposite resins, resulting in lower total strains ranging between 4.44% and 7.26% (Table 3). Although these values are higher in comparison to the commercial products Filtek^TM^Z350 XT and Tetric^®^ N-Ceram Bulk Fill (Table 3), the different monomer compositions and monomer to filler ratios may account for the observed tendencies [65]. Liu et al. found 5.32% shrinkage after inserting 20 wt% S.MPS nanofillers in dental composite resins, which is within the experimental values of Table 3 [62]. It is widely accepted that the incorporation of fillers in composite resins favors the decrease of polymerization shrinkage [73]. As a result, the substitution of TEGDMA content, well-considered to be responsible for shrinkage [74], with 22 wt% nanofiller in the synthesized nanocomposites stands for their decreased shrinkage in comparison to the starting Bis-GMA/TEGDMA resin. The aforementioned descending trend could be also ascribed to an increase of packing density due to the filler presence, accompanied by a limitation of polymer chain mobility [64] (Scheme 3). Moreover, the maximum values of 7.26% and 6.30% found for S.MPS and S.QAMTMSi containing dental resins could be associated with the highest final DC, as previously discussed. On the contrary, the lowest DC values measured for S.QAMTESi and S.QAPTESi composites account for their better dimensional stability after the photo-curing process. Based on this concept, the addition of S.QAPTESi retains the setting contraction rate at low levels during the whole polymerization reaction. 

### 3.7. Flexural Properties

**Figure 8:**
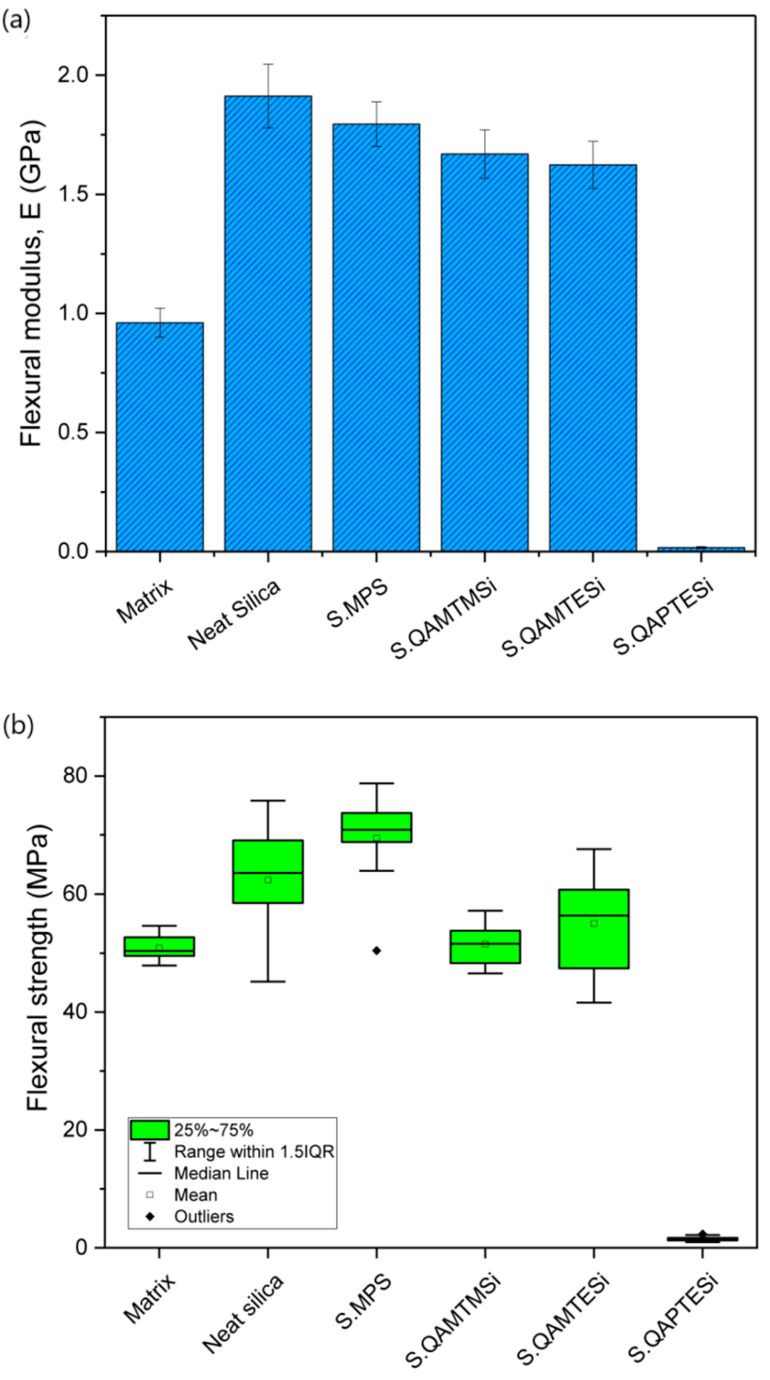
(**a**) Flexural modulus bar plots; (**b**) Flexural resistance box plots for pure Bis-GMA/TEGMA matrix and dental nanocomposite resins filled with different silica nanofillers.

Comparative charts of bare Bis-GMA/TEGDMA resin and dental nanocomposites concerning their flexural modulus and strength are given in Figure 8. Table 4 also contains the corresponding calculated mean or median values of flexural parameters in details, accompanied by their standard deviations (SD) or interquartile range (Q3–Q1) where applicable. Mean values corresponding to the commercially available products Filtek^TM^Z350 XT and Tetric^®^ N-Ceram Bulk Fill are also included for comparison reasons. The different chemical composition between the experimental and commercial dental composite resins may be mainly responsible for the observed variations in flexural properties. It is apparent that the stiffness was improved for the majority of nanocomposites in comparison to pure resin. More specifically, the flexural modulus was increased approximately by 99% for neat silica, 86% for S.MPS, 74% for S.QAMTMSi, and 69% for S.QAMTESi nanocomposite resins. Campos described Bis-GMA/TEGDMA based dental restorative composites exhibiting flexural modulus of 1.49–1.67 GPa when 50–70 wt% silanized silica was added [75]. Makvandi reported a modulus of elasticity around 2–2.5 GPa for nanocomposites containing 2.5–10 wt% quaternary ammonium silanizednanosilica [48]. In the current work, the calculated modulus values for the studied nanocomposites having 22 wt% of neat silica, S.MPS, S.QAMTMSi or S.QAMTESi ranged between 1.62–1.91 GPa, namely very close to the above literature data. The almost two-fold increment found for untreated silica could be probably attributed to the higher surface area of neat nanosilica rather than the other bulkier organomodified types of silica, resulting in higher surface energy in the hybrid matrix-filler interface. The derived interface adhesion between the matrix and nanofiller (Scheme 3) facilitates the stress transition from the flexible matrix to the stiffer nanosilica resulting in the aforementioned mechanical behavior [23,76,77,78]. In addition, the largest DC previously calculated for S.MPS nanocomposite resin probably led to the highest crosslinking density and finally to the best improvement of the modulus of elasticity in comparison to the other S.QASiC nanocomposites. On the other hand, the stereochemical hindrance of methacrylatedQAMTMSi molecules might be capable of limited mobility and the local copolymerization between S.QAMTMSi fillers, resulting in the formation of some agglomerates also confirmed by SEM findings (Figure 5c), and finally in lower flexural modulus than S.MPS composites, even if both S.QAMTMSi and S.MPS nanocomposite resins have almost the same final DC. Furthermore, the lower DC of S.QAMTESi in relation to S.QAMTMSi is associated with the observed marginally weaker stiffness.

In accordance to ISO 4049, the flexural strength requirement for a dental composite is around 80 MPa [80]. The flexural strength of most dental resins was clearly enhanced by the addition of 22 wt% silica nanoparticles, meeting the above standard prerequisite. It is worth pointing out that the nanocomposite containing S.MPS nanofillers yielded the maximum increment of strength (41%), followed by composites reinforced with neat nanosilica (26%), S.QAMTESi (12%), and S.QAMTMSi (2%) when compared to pure resin. Bindu reported almost 200% increase of flexural strength for dental restorative composites by increasing the 14 nm-silica content from 2 to 5 wt%, while the measured strength values varied from 26–88 MPa [81] which overlap the experimental data of the present dental nanocomposite resins. The different rigidity between polymer matrix and silica nanofiller is considered the main cause of fracture formation at the matrix–filler interface [78]. Either the competence of MPS, QAMTESi, and QAMTMSi molecules to react via their methacrylate groups with dimethacrylte monomers, creating strong coupling between the nanofiller and polymer matrix, or the increased surface energy of the interface due to the existence of extremely small untreated silica particles (12 nm) may contribute to the decrease of stress accumulation at the matrix–nanofiller interface, justifying the improved values of flexural strength in each case. The partial S.QAMTMSi particle interaction which is capable of agglomeration could affect the normal stress distribution at the interface, and thus moderates the induced resistance to flexural loadings.

However, the nanocomposite loaded with S.QAPTESi nanofiller disclosed a detrimental effect on its flexural properties. One possible explanation could be the occurrence of resin voids and large aggregates found on the composite’s surface by SEM (Figure 5e). This feature combination equals to structural defects which favor the stress concentration and crack propagation in high extent, causing mechanical failure even under early stage flexural loadings. Besides, the S.QAPTESi was found to contain the lowest effective chemical to physical absorbed silane ratio (Table 2). As a result, fewer methacrylated groups of silane covalently bonded to silica were available to copolymerize with monomers, leading to weaker interface adhesion between the S.QAPTESi filler and the Bis-GMA/TEGDMA matrix.

### 3.8. Compression Tests

**Figure 9:**
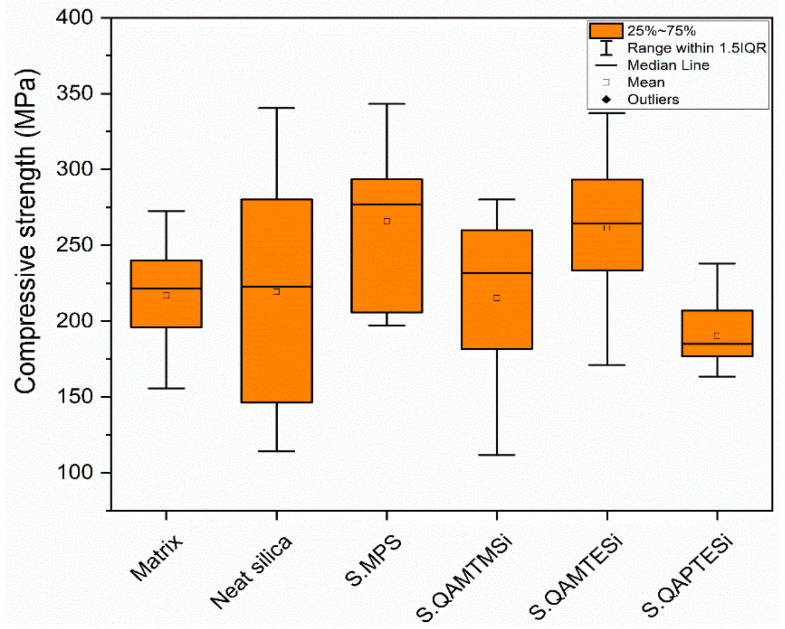
Compressive strength box plots for neat Bis-GMA/TEGMA matrix and experimental resins reinforced with diverse organomodified silica nanofillers.

Figure 9 shows the tendency of compressive strength between the obtained dental resins, while the corresponding values are listed in Table 4. Typical compressive strength values for conventional dental composite resins usually approach the range of 150–250 MPa [79,82], namely very close to those of the present experimental nanocomposites. It can be seen that the fracture resistance can be effectively enhanced mainly by the addition of S.MPS (25%) and S.QAMTESi (19%). The calculated strength for both of them was higher than that of 240.20–257.84 MPa found by Liu [62] after dental resin reinforcement with 10–30 wt% MPS modified nanosilica. A slight compressive strength augmentation was observed in the presence of neat silica and S.QAMTMSi fillers, while the ability of S.QAPTESi nanocomposite to withstand external forces was easily corrupted under lower exerted pressure. The improved performance against compressive loadings could be attributed to the filling of composite interstices with the particular type of silica rather than the polymer matrix according to SEM images (Figure 5a–d), which may lead to the increase of the nanofiller packing density, and eventually to the effective loading sustained by silica nanoparticles [62,64]. Except for the different extent of dispersion of each organomodified silica in the organic matrix (Figure 5) and degree of conversion, a possible diverse orientation (perpendicular or horizontal) of silane molecules on the silica surface [23] could also account for the particular mechanical performance as described above. However, the structural discrepancies of S.QAPTESi nanocomposite did not allow the dissipation of the tensile forces acting on compression test.

## 4. Conclusions

New methacrylated quaternary ammonium silanes were successfully synthesized through a variant of theMenschutkin reaction and subsequently used to modify silica nanoparticles. The targeted chemical structures of the obtained organosilanes were confirmed by means of ^1^H-NMR and FTIR spectra. The combination of FTIR and TGA findings verified not only the surface modification of silica, but also highlighted the influence of the specialized ammonium silane molecule on the efficacy of the silanization process, determining that methoxy substituents favor the chemical absorption of silane on nanosilica’s surface. The different types of organomodified silica were then effectually inserted in Bis-GMA/TEGDMA based matrix resulting in morphological characteristics dominated by well-dispersed nanoclusters as it was observed by SEM for the majority of the synthesized dental nanocomposite resins. Polymerization kinetics was mainly characterized by the occurrence of the intensified gel-effect phenomenon in the presence of nanosilica modified with the smaller silane molecules (S.MPS, S.QAMTMSi, S.QAMTESi), leading to an expected increment in the DC value for nanocomposite containing the shorter molecule of S.QAMTMSi (59.16%), namely very close to that of the S.MPS nanocomposite (59.56%) which was used as control material. The addition of silica nanoparticles verified the anticipated reduction of the polymerization shrinkage for all composites due to the presence of silica, even if variations of setting contraction were correlated to the specific silane structure. More specifically, S.QAMTESi nanocomposite exhibited the lowest shrinkage strain (4.44%) which can be ascribed to the relatively low DC (55.67%). Most dental nanocomposite resins exhibited improved mechanical properties. In particular, they were found to be dependent on the degree of conversion and the extent of nanofiller dispersion in the polymer matrix. Although the highest DC found for S.MPS resulted in the maximum flexural and compressive strength, the partial S.QAMTMSi particle agglomeration can moderate the flexural properties despite the corresponding improved DC value. The findings of the study could be considered in the light of some limitations, such as the evaluation of antibacterial properties and cytotoxic effects of the current nanocomposites. These limitations could be addressed in the future, thus contributing to a holistic approach towards the modern designation of multifunctional dental materials meeting the requirements of clinical practice. In each case, the present research provides new data in the field of dental composite resins via the utilization of novel multifunctional nanoparticles, thus taking a step forward for the pursuit of scientific knowledge.

## Figures and Tables

**Table 1:** Chemicals utilized for the modification of nanosilica and the synthesis of dental nanocomposite resins.

Name	Specifications	Supplier
2,2-Bis[p-(2′-hydroxy-3′-methacryloxypropoxy)phenylene]propane (Bis-GMA)		SIGMA-ALDRICH CHEMIE GmbH (Steinheim, Germany)
Triethylene glycol dimethacrylate (TEGDMA)	95%	
Cyclohexane	≥99.5%	
Silica nanopowder	99.8%, average diameter: 12 nm, specific surface area (BET): 200 ± 25 m^2^·g^−1^	
(3-chloropropyl)triethoxysilane (3CPTESi)	95%	
Camphorquinone	98%	J&K Scientific GmbH (Pforzheim, Germany)
2-(dimethylamino)ethylmethacrylate (DMAEMA)	99%	
3-(trimethoxysilyl)propyl methacrylate (γ-MPS)	98%	
(chloromethyl)trimethoxysilane (CMTMSi)	96%	
(chloromethyl)triethoxysilane (CMTESi)	97%	abcr GmbH (Karlsruhe, Germany)
Propylamine	≥99.0%	Merck KGaA (Darmstadt, Germany)
Methanol	≥99.8%	Honeywell/Riedel-de-Haën (Seelze, Germany)

**Table 2:** Percentage (wt%) of each silane attached on the silica surface determined by TGA method.

Organomodified Nanosilica	Total (wt%)	Physically Absorbed			Chemically Absorbed			Effective Functional Ratio
		wt%	μmol·m^−2^	Molecules·nm^−2^	wt%	mmol·m^−2^	Molecules·nm^−2^	
S.MPS	5.21	1.10	0.22	0.13	4.11	0.83	0.50	3.74
S.QAMTMSi	8.98	3.55	0.54	0.33	5.43	0.83	0.50	1.53
S.QAMTESi	9.77	4.50	0.61	0.37	5.27	0.71	0.43	1.17
S.QAPTESi	6.94	3.50	0.44	0.27	3.44	0.43	0.26	0.98

**Table 3:** Curing kinetics and polymerization shrinkage data calculated for Bis-GMA/TEGDMA matrix and dental nanocomposite resins. Literature data for typical commercially available dental resins are also included [65].

Material	Final DC (%)	Total Strain (%)
Organic matrix	54.20	8.84 ± 0.26
Neat silica nanocomposite	54.98	4.67 ± 0.70
S.MPS nanocomposite	59.51	7.26 ± 0.46
S.QAMTMSi nanocomposite	59.16	6.30 ± 0.70
S.QAMTESi nanocomposite	55.67	4.44 ± 0.98
S.QAPTESi nanocomposite	36.17	4.82 ± 0.44
Filtek^TM^Z350 XT (3M ESPE, St. Paul, MN, USA) [65]	50.96	1.66 ± 0.15
Tetric^®^ N-Ceram Bulk Fill (Ivoclar-Vivadent, Schaan, Liechtenstein) [65]	49.50	1.36 ± 0.08

**Table 4:** Mean values ± standard deviation (SD) of flexural modulus and median values ± inter-quartile range (IQR) of flexural strength and compressive strength for Bis-GMA/TEGDMA matrix and dental nanocomposite resins. Literature data for typical commercially available dental resins are also included.

Material	Flexural Modulus (GPa) ^1^	Flexural Strength (MPa) ^1^	Compressive Strength (MPa)
Organic matrix	0.96 ± 0.06 ^a,b,c,e,g^	50.37 ± 3.16 ^j,k,l,m^	221.48 ± 43.95
Neat silica nanocomposite	1.91 ± 0.13 ^a,c,e,g^	63.54 ± 10.56 ^j,l,n,o^	222.86 ± 133.86
S.MPS nanocomposite	1.79 ± 0.09 ^b,d,f,h^	70.86 ± 4.94 ^k,m,p,q,r^	276.72 ± 87.82
S.QAMTMSi nanocomposite	1.67 ± 0.10 ^c,d,g^	51.58 ± 5.51 ^n,p,r^	231.81 ± 78.41
S.QAMTESi nanocomposite	1.62 ± 0.10 ^e,f,i^	56.36 ± 13.35 ^q,s^	264.17 ± 60.03
S.QAPTESi nanocomposite	0.02 ± 0.00 ^g,h,i^	1.57 ± 0.40 ^l,m,o,r,s^	184.87 ± 30.17
Filtek^TM^Z350 XT (3M ESPE, St. Paul, MN, USA) [65,79]	9.13 ± 0.66	80.52 ± 15.88	226.92 ± 6.64
Tetric^®^ N-Ceram Bulk Fill (Ivoclar-Vivadent, Schaan, Liechtenstein) [65,79]	7.05 ± 0.60	60.37 ± 11.05	214.28 ± 3.67

^1^ The groups with the same superscript letters exhibit statistically significant differences (*p* < 0.001).

## Data Availability

The data presented in this study are available on request from the corresponding author.
